# Taste Hedonics Influence the Disposition of Fat by Modulating Gastric Emptying in Rats

**DOI:** 10.1371/journal.pone.0090717

**Published:** 2014-03-03

**Authors:** Katsuyoshi Saitou, John N. Lees, Michael G. Tordoff

**Affiliations:** 1 Monell Chemical Senses Center, Philadelphia, Pennsylvania, United States of America; 2 Health Care Food Research Laboratories, Kao Corporation, Tokyo, Japan; University of Tokyo, Japan

## Abstract

We investigated how preferred and nonpreferred tastes influence the disposition of fat. Adult male Sprague Dawley rats were infused with 5 ml of 20% intralipid through an intragastric catheter and with 0.3 ml of a taste solution through an intraoral catheter. At 120 min postinfusion, plasma concentrations of fat fuels (triglycerides and non-esterified fatty acids) were either unchanged or slightly higher after rats tasted a preferred sweet taste solution (0.125% saccharin +3% glucose) than after they tasted water. They were markedly lower after rats tasted a non-preferred solution–either a bitter solution (0.15% quinine hydrochloride) or a sweet solution that had previously been the conditioned stimulus for lithium-induced taste aversion. The distribution of ^14^C-triolein mixed with the gastric load was determined at 4 h postinfusion. Rats that received a non-preferred bitter taste had significantly more ^14^C remaining in the stomach than did those that received a preferred sweet taste. These results suggest that taste hedonics–either unconditioned or conditioned aversive tastes–influence fat disposition by altering gastric emptying.

## Introduction

Elevated plasma triglyceride levels have well-established links with chronic diseases such as obesity, insulin resistance and cardiovascular disease [1], [2]. There is growing interest in the function of triglycerides during the postprandial state [3]–[6] in large part because postprandial hypertriglycemia is a risk factor for cardiovascular disease [3], [4]. One mechanism involves elevated triglycerides after a meal recruiting monocytes and inflammatory signaling molecules that eventually lead to atherosclerosis [5], [6]. However, there is need for a comprehensive understanding of what regulates postprandial fat disposition.

Oral sensation, especially taste perception, plays a primary role in food selection [7]–[12] but also guides the disposition of ingested nutrients. Sweet taste and other orosensations elicit gastric emptying [13], digestive enzyme secretion [14], and insulin release [15]–[17]. These physiological responses, which are commonly referred to as cephalic phase responses [18], [19], prepare the gut and other organs for the approaching absorption and distribution of nutrients.

Several lines of evidence suggest that orosensation modifies fat disposition. Oral fat stimuli increase plasma triglyceride concentrations in both animal and human studies: In rats, oral exposure to corn oil or sweet taste leads to a more prolonged elevation of plasma triglycerides relative to oral water or no taste exposure [20]. In humans, oral fat elicits a rise of plasma triglycerides at two different time points; a small spike at ∼1 h after fat loading that is derived from intracellular lipids in enterocytes, followed at ∼4 h by a prolonged elevation of triglycerides [21], [22]. Tasting and expectorating is sufficient to augment the rise in postprandial triglycerides by influencing both the production of chylomicrons and the metabolism of very low density lipoproteins [23].

The basic findings that oral stimuli influence fat trafficking have been replicated and extended [24]–[26], but there has been little attention to whether the chemical or hedonic properties of taste are responsible. Preference (i.e., liking) is an important aspect of taste as well as quality (i.e., sweet, bitter, salty, fatty, etc.). The purpose of the present study was to examine how preferred and nonpreferred tastes influence the disposition of fat. To this end, we infused fat directly into the stomach of rats with implanted intragastric and intraoral catheters. Orosensation was manipulated by infusing preferred or non-preferred taste compounds into the oral cavity. The disposition of infused fat was observed by evaluating blood triglyceride and fatty acid concentrations, and the distribution of radioactive ^14^C-fat mixed with the gastric load.

## Materials and Methods

### Animals & Maintenance

Male Sprague–Dawley rats (weighing 351–375 g; Charles River Laboratories, Raleigh, NC) were housed individually in stainless steel cages at 22°C on a 12∶12-h light-dark cycle (lights on at 06∶00). The rats had free access to AIN-76A diet and deionized water, unless otherwise mentioned. The experiment protocol was approved by the Monell Chemical Senses Center Institutional Animal Care and Use Committee [protocol no. 1149].

### Materials

As taste stimuli, we used a “sweet solution” consisting of a mixture of 0.125% saccharin and 3% glucose (both Sigma-Aldrich, St Louis, MO) or a “bitter solution” consisting of 0.15% (0.0038 M) quinine hydrochloride (Sigma-Aldrich). The saccharin-glucose mixture is avidly ingested by rats [12]; the 0.15% quinine is strongly disliked–tasting it elicits negative hedonic responses (i.e., gapes and chin rubs) and it is almost completely avoided in two-bottle preference tests [7]. As an intragastric fat load, 20% intralipid was purchased from Sigma-Aldrich (Cat. No. I-141). Radioactive ^14^C-triolein was purchased from American Radiolabeled Chemicals Inc (St Louis, MO) and stored at −20°C until use.

The following enzymatic colorimetric kits or ELISA kits were used for the assay of blood components; triglycerides, ketones, glycerol and glucose from Cayman Chemical Co. (Ann Arbor, MI); non-esterified fatty acid from Wako Diagnostics (Richmond, VA); insulin from Alpco Diagnostics (Windham, NH); total GIP, total GLP-1 and leptin from Millipore (Billerica, MA); peptide YY and cholecystokinin from Phoenix Pharmaceuticals (Belmont, CA).

### Surgery

At least 5 days after arrival, rats were surgically implanted with an intragastric catheter and an intraoral cannula. The rats were anesthetized with an intraperitoneal injection of 1 ml/kg of the following mixture: ketamine (4.28 mg/ml; Ketaset, Fort Dodge Animal Health, Fort Dodge, IA), xylazine (0.86 mg/ml; AnaSed, Lloyd Laboratories, Shenandoah, IA), and acepromazine (0.14 mg/ml; Aceproject, Butler, Bublin, OH). For the intragastric surgery, a midline incision was made, the stomach was gently retracted, and a Silastic catheter (0.64-mm ID, 1.19-mm OD) was inserted ∼1 cm through a hole poked with an 18-gauge needle through the glandular portion of the stomach. The catheter was fixed to the gastric wall with 2–0 silk suture. The distal end of the catheter was passed under the skin and exteriorized at the back of the neck. It was glued to a 1-cm square piece of Marlex mesh that was mounted under the skin to anchor it, and the exteriorized portion was sheathed in Tygon tubing to protect it from being bitten.

The intraoral cannula consisted of polyethylene-90 tubing (Warner Instruments, Hamden, CT) with one end flared and fixed with a small Teflon disc (6-mm diameter, 0.8-mm thickness). The cannula was inserted into the cheek immediately lateral to the first molar. The Teflon disk was placed so as to rest against the inside of the cheek, and the other end of the cannula was exteriorized at the same position as the gastric catheter and fixed there.

Shortly after surgery, and again on the following day, the rats were treated with antibiotics (Triple Antibiotic Ointment, Medique, Fort Myers,FL) to prevent infections and with buprenorphine hydrochloride (Buprenex, Reckitt Benckiser Pharmaceuticals Inc., Richmond, VA) to alleviate discomfort. The patency of the intragastric catheter and intraoral cannula was checked every 2 or 3 days by flushing saline; any rat with a blocked or broken catheter or cannula was excluded from the experiments.

After at least 7 days to recover from surgery, rats were given three training sessions (one a day) in order to habituate them to the test procedures. To do this, two intraoral infusions, one of 0.5 ml water and one of 0.5 ml sweet solution, were introduced into the oral cavity in a randomized order, with a 5-min interval between them. These training sessions were conducted between 09∶00 and 12∶00 (light period).

### Test Procedure

#### Experiment 1

Before the test, some rats were subjected to procedures designed to induce a conditioned taste aversion to the sweet solution (*n* = 10). To do this, 0.5 ml of sweet solution was infused intraorally and immediately followed by an intraperitoneal injection of 4 ml/kg•BW of LiCl (20 mg/ml; Sigma-Aldrich) as the malaise-inducing agent (conditioned group). The same volume of isotonic saline was injected into rats of the unconditioned group (*n* = 9). This injection procedure was repeated after 3-days so that each rat received two taste aversion conditioning trials.

The test was started 3 days later. All rats received two tests: one with the sweet solution and one with water presented orally as a control. The order of these tests was randomized (crossover design) and there was a 1-week interval between them. On each test day, the rats were deprived of food beginning 1 h before testing began until the end of the test session. The rats were infused with 5 ml of 20% intralipid through the gastric catheter at a rate of 1 ml/min using a Sage syringe pump (model 351; Orion Research Inc., Cambridge, MA). Immediately after the infusion, the rats were infused with 0.3 ml water or the sweet solution through the intraoral cannula. At 15 min before (−15 min) and then at 30, 120 and 240 min after the gastric infusion, blood was collected from the tip of the tail of awake rats into heparinized capillary tubes (Fisher Scientific, Pittsburgh, PA). After the ∼140 µl sample was withdrawn, one end of the capillary tube was sealed with Critoseal (McCormick Scientific, St. Louis, MO). Within no more than 5 min, the whole blood was centrifuged for 2 min (IEC MB microhematocrit centrifuge; International Equipment Co., Needham Heights, MA), and plasma collected. The plasma samples were used for the assays on the same day as they were prepared.

**Figure 1:**
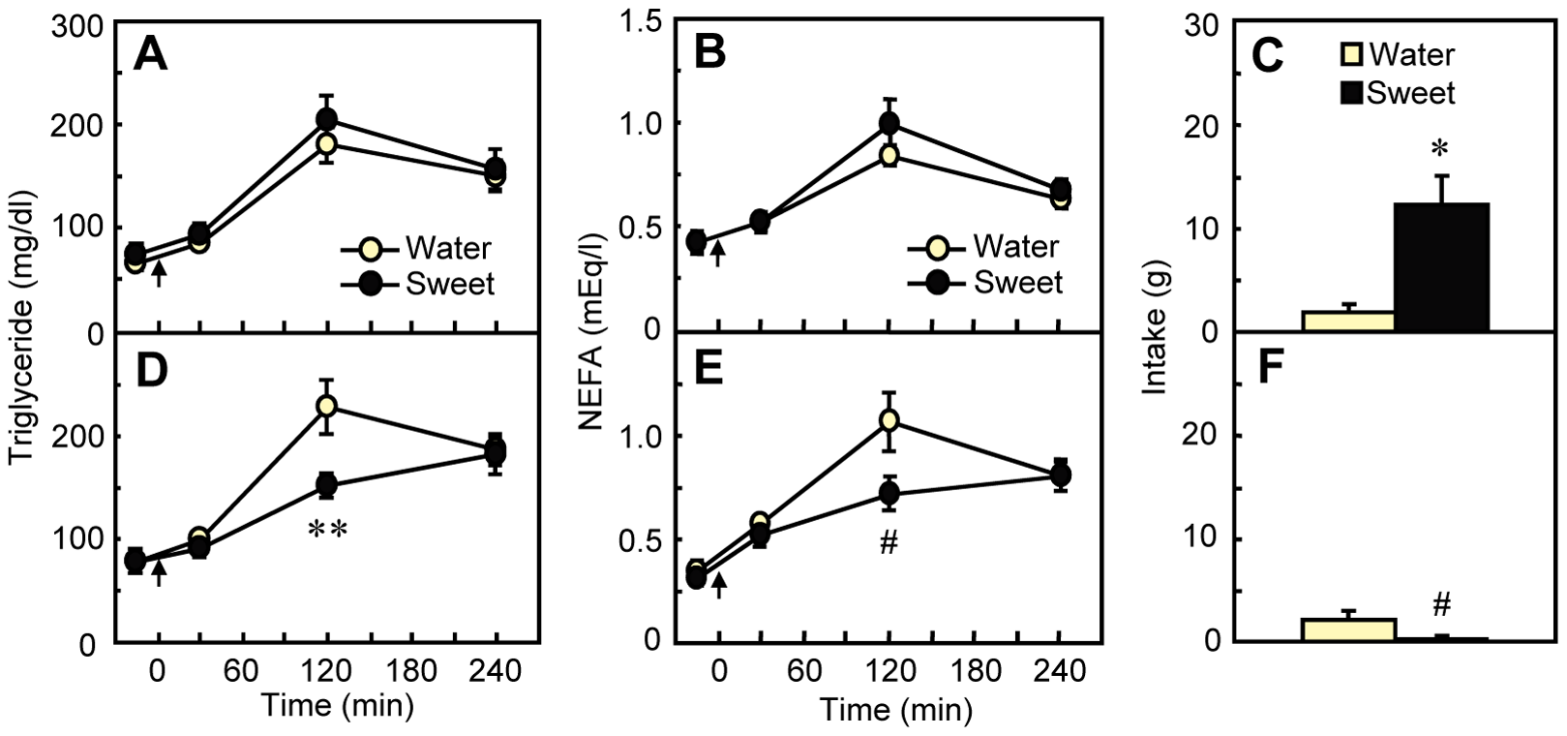
Comparison of the effects of sweet taste on blood fat levels between unconditioned rats and rats conditioned to show aversion to the taste. (A–C) upper panels: unconditioned rats (*n* = 9); (D–F) lower panels: conditioned rats (*n* = 10). Plasma concentrations of (A, D) triglyceride and (B, E) NEFA were measured after the gastric infusion of 20% intralipid (started at 0 min; arrow) and the following oral infusion of 0.3 ml of water or sweet solution. (C, F) Intake of sweet solution in 2-bottle choice test at the end of the experiment showed conditioning had occurred successfully. Values are means ± S.E.M. *P<0.05, **P<0.01 by paired t-test.

To verify conditioning had occurred successfully, at the end of the experiment two-bottle choice tests were conducted. To do this, the rats were first deprived of food and water for 5 h, and then given two drinking bottles, with one containing water and one sweet solution for 1 h. Intakes were measured by weighing the bottles (±0.1 g) before and after the presentation. During this test, the unconditioned rats drank 12.1±2.7 ml sweet solution and 1.9±0.7 ml water (87% preference); the conditioned group drank 0.2±0.1 ml sweet solution and 2.0±0.8 ml water (9% preference). Thus, the conditioning procedure was successful (Figure 1C, 1F).

#### Experiment 2

Exposure to a preferred sweet taste had no effect on blood fat fuels in *Experiment 1* (see Results, below). This appeared at least superficially discrepant with earlier work, In particular, using procedures similar to ours, Ramirez [20] showed that tasting saccharin elevated blood fat concentrations, particularly when the sweet taste had previously been paired with an intragastric fat load. A methodological concern was that in our Experiment 1 rats received saline injections during conditioning procedures. This additional handling might potentially influence the rats’ subsequent responses. We therefore repeated the blood fat analysis test used in *Experiment 1* in 12 naïve rats, except this cohort did not receive any conditioning procedures. The rats received a gastric infusion of 5 ml of 20% intralipid followed immediately by 0.3 ml intraoral water or sweet solution. Blood samples for analysis of triglycerides and fatty acids were collected at −15, 30, 120 and 240 min.

#### Experiment 3

Several studies show that sweet taste receptors are present in the intestines and are functional [28], [29]. To evaluate their potential contribution to the fat disposition observed in *Experiment 1* and *2,*, in *Experiment 3,* the taste solution was infused intragastrically in 11 rats. Intralipid was delivered in the same manner as in *Experiments 1* and *2* (i.e., 5 ml of 20% intralipid at 1 ml/min) and then either 0.3 ml water or sweet solution was infused through the intragastric catheter over 20 sec. Blood was collected from the tail at −15, 30, 120 and 240 min. All rats received two tests: one with the sweet solution and one with water.

#### Experiment 4

In this experiment, we determined the effect of an unconditioned avoided taste on fat disposition. Bitter quinine hydrochloride solution was used as a taste solution. The procedure was the same as for *Experiment 1* and *2*: Immediately after the intragastric infusion of 5 ml of 20% intralipid, the rats (*n* = 10) were infused with 0.3 ml water or the bitter solution through the intraoral cannula. Blood was collected from the tail and used for the assays.

#### Experiment 5

**Figure 2:**
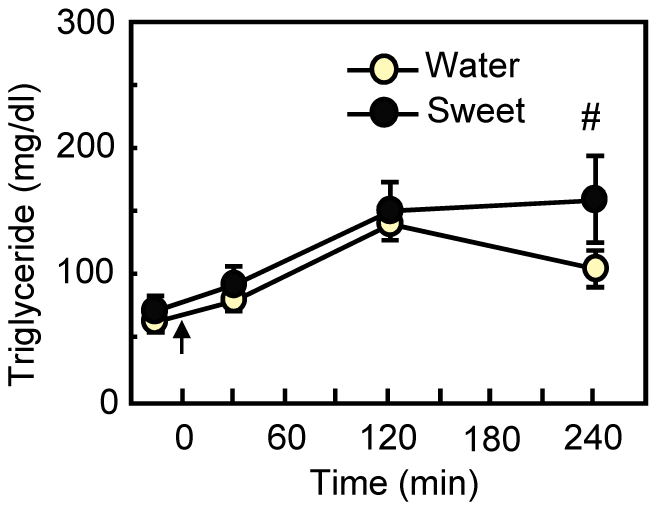
Influence of preferred sweet taste on plasma triglyceride concentration in untreated rats. The gastric infusion of 20% intralipid was started at 0 min (arrow). Immediately after the infusion, the rats (*n* = 12) were given 0.3 ml of water or sweet solution through the intraoral cannula. Values are means ± S.E.M. #*P*<0.1 by paired t-test.

In this experiment, the organ distribution of fat was traced by the recovery of radioactivity from intragastrically infused ^14^C-triolein. We assessed tissue radioactivity in the gastrointestinal tract and in several organs at 4 h after fat infusion, the time at which the largest effect of sweet taste was observed in earlier experiments (Figure 2). One hour before the experiment (at 09∶30–10∶00), each rat was moved to a plastic cage (28 cm×45 cm×20.5 cm) with woodchip bedding. It was infused with 1.0 µCi of ^14^C-triolein in 5 ml intralipid into the stomach at a rate of 1 ml/min. Immediately after that, it was given 0.3 ml of water (*n* = 8), sweet taste solution (*n* = 8) or bitter taste solution (*n* = 7) through the intraoral cannula over 20 sec. At 4 h after the infusion, it was deeply anesthetized with isoflurane (AErrane; Baxter, Deerfield, IL) and blood was collected by cardiac puncture. The blood was transferred into a 1.5-ml Eppendorf tube and allowed to clot at room temperature for 30 min. Serum was prepared by centrifugation at 3000 g for 15 min at 4°C. The serum was used for the measurement of radioactivity and the assay of blood components.

After the cardiac puncture, each rat was dissected and pertinent organs (stomach, small intestine, colon, heart, liver and kidney) and tissues (femoris muscle and epididymal fat) were excised. The stomach, small intestine and colon were opened and their contents were collected by washing their inner walls three times with 3 ml of phosphate-buffered saline (Mediatech Inc., Herndon, VA). The collected gut contents were weighed and homogenized. Other organs and tissues were weighed and homogenized in 10 ml of phosphate-buffered saline. One milliliter aliquots of each homogenate were added to 10 ml scintillation fluid (Scintiverse; Fisher Scientific), and radioactivity was measured using a Packard Instruments beta scintillation counter to determine tissue uptake. Values were expressed as a percentage of the total radioactivity infused.

### Statistical Analysis

Differences between rats given different oral treatments were assessed using analyses of variance with factors of Taste (water, sweet and/or bitter) and Time (if measurements were made at more than one time). Differences between the treatments at particular times were assessed using paired t-tests or Fisher’s LSD post hoc tests (when comparisons of more than 3 groups were required). Results are expressed as means ± S.E.M.

## Results

### Experiment 1: Hedonically Aversive Taste Decreases Blood Fat Concentrations

In *Experiment 1*, we examined whether sweet taste influenced the disposition of intragastrically infused fat. The hedonic value of the taste was manipulated by eliciting a conditioned taste aversion to sweetness in one group of rats. In the unconditioned group (*n = *9), sweet taste had no significant effects on blood triglycerides or NEFA levels relative to the water control condition (Figure 1A, 1B). In the conditioned group (*n* = 10), on the other hand, sweet taste significantly decreased blood triglycerides [main effect of Taste, *F*_1,9_ = 8.39, *P* = 0.018, Taste×Time interaction; *F*_3,27_ = 4.00, *P* = 0.018; Figure 1D]. In addition, NEFA levels were also decreased by the sweet taste in a similar pattern [main effect of Taste; *F*_1,9_ = 2.37, *P* = 0.158, Taste×Time interaction; *F*_ 3,27_ = 4.00, *P* = 0.018; Figure 1E]. For both fat fuels, the difference was evident at 120 min postinfusion, but not at earlier or later times.

### Experiment 2: Replication that a Preferred Sweet Taste does not Significantly Influence Blood Fat Concentrations

Replicating the results of *Experiment 1*, animals in this experiment also did not display a significant influence of sweet taste on triglyceride concentrations [main effect of Taste; *F*_ 1,11_ = 2.50, *P* = 0.142, Taste×Time interaction; *F*_ 3,33_ = 1.95, *P* = 0.140; Figure 2]. There was a tendency for sweet taste to elevate triglycerides at 240 min postinfusion, but this was nonsignificant even by paired t-test (*P* = 0.058).

### Experiment 3: Gastrointestinal Sweet Taste Infusions do not Influence Blood Fat Concentrations

**Figure 3:**
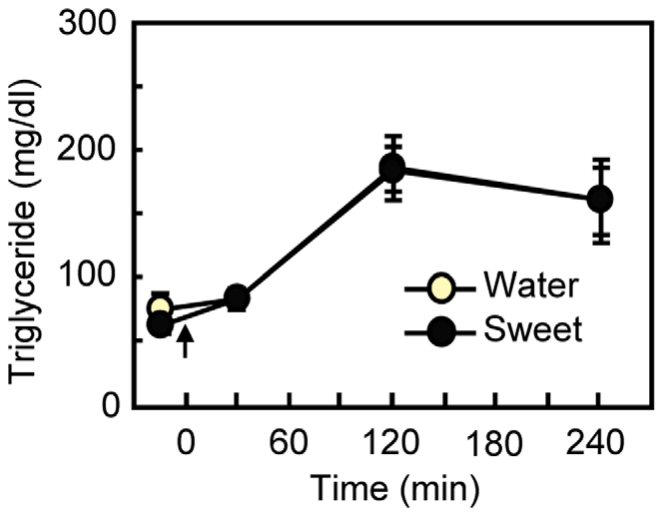
Influence of gastric infusion of sweet taste on plasma triglyceride concentration. The gastric infusion of 20% intralipid was started at 0 min (arrow). Immediately after the infusion, the rats (*n* = 11) were given 0.3 ml of water or sweet solution through the intragastric catheter. There was no significant difference in triglyceride levels between sweet and water infusion. Values are means ± S.E.M.

Infusion of the sweeteners into the stomach had no effect on blood triglycerides [main effect of Taste; *F*_1,10_ = 0.07, *P* = 0.801, Taste×Time interaction; *F*_ 3,30_ = 0.24, *P* = 0.870; Figure 3].

### Experiment 4: Bitter Taste Decreases Blood Fat Levels

**Figure 4:**
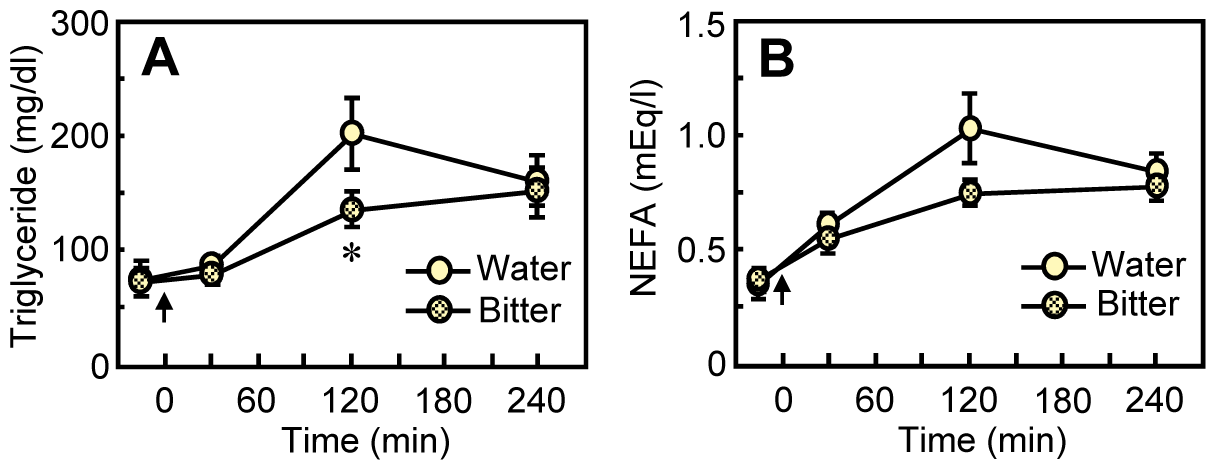
Influence of aversive bitter taste on on blood fat levels. The gastric infusion was started at 0(arrow). Immediately after the infusion, the rats (*n* = 10) were given 0.3 ml of water or bitter solution through the intraoral cannula. Values are means ± S.E.M. #*P*<0.1, *P<0.05, **P<0.01 by paired t-test.

In *Experiment 4*, we determined whether an innately aversive bitter quinine hydrochloride taste solution [9], [10] influenced fat disposition. Relative to water taste, bitter taste decreased blood triglyceride levels significantly [main effect of Taste; *F*_ 1,9_ = 7.25, *P* = 0.025, Taste×Time interaction; *F*_ 3,27_ = 3.81, *P = *0.021; Figure. 4A] and tended to decrease NEFA levels [main effect of Taste; *F*_ 1,10_ = 2.21, *P = *0.171, Taste×Time interaction; *F*_ 3,30_ = 2.39, *P* = 0.091; Figure 4B] in a similar pattern to the conditioned aversive sweet taste (*Experiment 1*).

### Experiment 5: Taste Influences Fat Disposition by Altering Gastric Emptying

**Figure 5:**
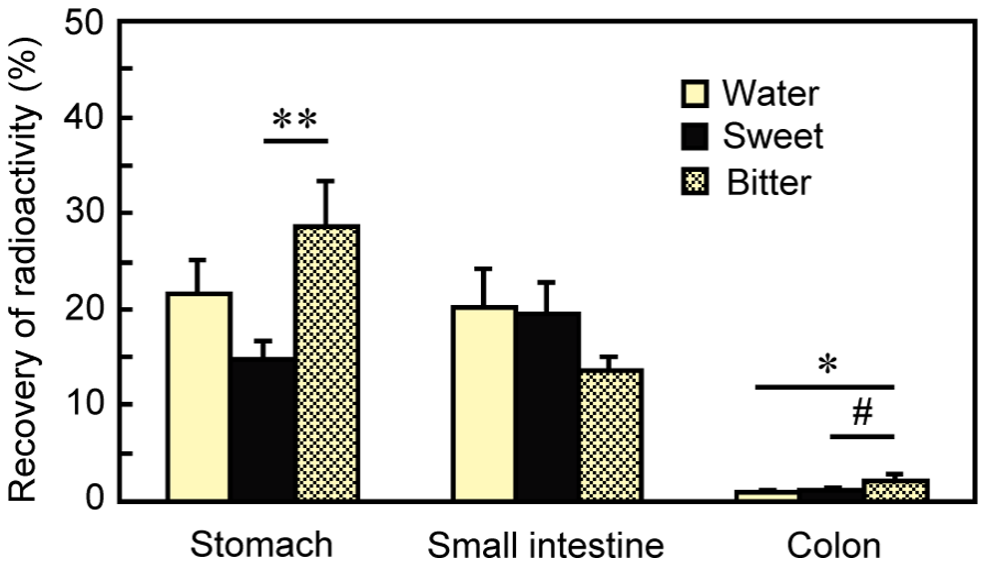
Gut content radioactivity recovered at 4 h after intragastric infusion of ^14^C-triolein. Rats were given intraoral infusions of water (*n* = 8), sweet (*n* = 8) or bitter taste stimuli (*n* = 7) immediately after intragastric infusion of ^14^C-triolein in intralipid. Values are means ± S.E.M. #*P*<0.1, *P<0.05, **P<0.01 by post-hoc t-test.

In *Experiment 5*, we compared the tissue distribution of ^14^C-triolein, and a panel of blood fuels and hormones at 4 h after rats received oral exposure to water, sweet solution, or bitter solution. There were large and significant differences in stomach contents [*F*_ 2,20_ = 4.11, *P* = 0.032; Figure 5]. Rats exposed to the bitter taste solution had significantly more–about twice as much–radioactivity in the stomach than did rats exposed to the sweet taste solution. There were small, albeit significant differences among the three groups in radioactivity in the colon (Figure 5). The distribution of radioactive fat in the other tissues did not differ (Table 1).

**Table 1:** Influence of taste on recovery of radioactivity in several body tissues at 4 h after intragastric infusion of ^14^C-triolein in intralipid.

	Taste stimuli		
Component	Water	Sweet	Bitter
Blood (Serum)	0.08±0.01	0.11±0.01	0.08±0.02
Heart	0.17±0.02	0.15±0.02	0.15±0.03
Liver	0.21±0.03	0.20±0.02	0.16±0.03
Kidney	0.09±0.01	0.07±0.01	0.08±0.01
Muscle tissue	0.06±0.01	0.06±0.01	0.05±0.01
Fat tissue	0.08±0.02	0.10±0.02	0.08±0.02

Values are means ± S.E.M (*n* = 7–8) percentage per 1 g tissue weight of the total amount of radioactivity administered.

In this experiment, there were significant effects of the sweet taste on triglyceride concentrations [*F*_ 2,20_ = 3.51, *P* = 0.049]. Sweet taste significantly increased blood triglycerides compared with water (*P* = 0.017, post-hoc test) but not bitter taste (*P* = 0.101). Blood hormone concentrations were unaffected by taste, with the exception that bitter taste decreased blood GLP-1 levels relative to water (*P* = 0.025) and sweet taste (*P* = 0.036; *F*_ 2,20_ = 3.57, *P* = 0.047; Table 2).

**Table 2:** Influence of taste on the concentration of blood components at 4 h after rats received an intragastric intralipid infusion.

	Taste stimuli		
*Component*	Water	Sweet	Bitter
*TG, mg/dl*	76±14	146±24 a	98±19
*NEFA, mEq/l*	0.51±0.06	0.68±0.06	0.55±0.09
*Ketone, mM*	0.29±0.04	0.37±0.05	0.33±0.07
*Glucose, mg/dl*	100±6	108±6	102±12
*Insulin, ng/ml*	2.03±0.56	1.78±0.33	2.12±0.42
*GIP, ng/ml*	227±28	210±31	184±22
*GLP-1, pM*	24.8±3.5	24.0±3.9	12.8±2.9 a b
*Leptin, ng/ml*	6.07±1.95	8.60±2.03	7.95±2.69
*PYY, ng/ml*	0.75±0.11	0.66±0.09	0.92±0.12
*CCK, ng/ml*	1.12±0.09	1.03±0.12	1.29±0.14

Values are means ± S.E.M. (n = 7–8).

a significantly different from Water group (*P*<0.05, by post-hoc test),

b significantly different from Sweet group (*P*<0.05). Abbreviations: *TG* triglyceride, *NEFA* non-esterified fatty acid, *GIP* glucose-dependent insulinotropic peptide, *GLP-1* Glucagon-like peptide-1, *PYY* Peptide YY, *CCK* Cholecystokinin.

## Discussion

We demonstrate here that the hedonic value of a taste can affect the disposition of an intragastric fat load. When accompanied by the taste of water, intragastric fat infusions transiently elevated blood triglycerides and NEFAs, with a peak occurring at about 120 min postinfusion (Figure 1–4). Identical fat infusions accompanied by hedonically negative tastes–either an innately avoided bitter taste (Figure 4) or a sweet taste that had been associated with malaise (Figure 1)–increased blood triglycerides and NEFAs significantly less. A hedonically positive sweet taste had more ephemeral effects: Relative to the taste of water, sweet taste increased blood fat levels significantly in one experiment (Table 2), had a tendency for an effect in this direction in another (Figure 2), and produced no difference in a third (Figure 1).

The decrease in blood fat concentrations produced by exposure to unpleasant taste is most likely secondary to altered absorption processes, especially gastric emptying. Rats that tasted quinine after a fat load had markedly more radioactive fat label remaining in the stomach 4 h later than did rats that tasted water or a sweet solution (Figure 5). The action of unpleasant taste to retard gastric emptying is consistent with other studies. For example, Yamamoto et. al. showed that quinine-containing bitter mash stays longer in the stomach of rats than does unadulterated mash [13], and Wicks et. al. demonstrated that bitter taste delays gastric emptying in humans [32]. This also makes teleological sense: Unpleasant taste normally signifies a food that is toxic. Slowing gastric emptying reduces the rate of absorption of the toxin and thus minimizes its blood concentrations.

There are several potential explanations for why sweet taste produced only ephemeral effects on the disposition of a gastric fat load. These include (i) methodological factors, in particular, the times we sampled blood or/and the deprivation condition we posed on rats may be important. Oral stimuli mobilize the endogenous fat stored in enterocytes and release it into the circulation rapidly [21], [33]; it is unlikely that we captured this considering the relatively late time points at which we observed effects (i.e., 2 or 4 h after taste exposure). (ii) Physiological factors may influence the appearance of fat in the blood. For example, the increased rate of fat absorption caused by sweet taste might be accompanied by increased tissue uptake of fat (perhaps mediated by insulin), leading to stable blood fat concentrations despite increased turnover. (iii) Hedonic factors may be involved. Sweet and bitter tastes are at opposite ends of a palatability continuum; the “control” water taste may fall closer to the sweet end than bitter end of the continuum, making the sweet-water contrast smaller than the bitter-water contrast. Indeed, water is sometimes considered to be sweet [34]. (iv) It may be that sweet taste has little effects on fat disposition but instead prepares the body to metabolize carbohydrates [35]. As shown by Ramirez [20], the association of sweet taste with an intragastric fat load may be necessary for eliciting the maximum effects on fat disposition. It will require additional research to assess these possibilities. But whatever the mechanism, it is clear that the preferred sweet taste never decreased blood fat levels, which contrasts with the effects of nonpreferred tastes.

Taste receptors are present in the gastrointestinal tract where they can initiate hormonal and neural responses to chemical stimuli [27]–[31]. In fact, Janssen et. al. [30] and Glendinning et. al. [31] have demonstrated that intragastric infusion of a bitter taste can delay gastric emptying. In our study, direct intragastric infusion of sweet taste solution did not show any effects on fat disposition (Figure 3) although the same infusion given orally was effective (Figure 1). We suspect that the 0.3-ml volume of taste solution we used in this study was too small to activate the gut taste system, while being easily sufficient to evoke oral sensation [7].

Further studies are needed to elucidate the physiological mechanisms involved in the modification of fat disposition by taste. Like many other cephalic phase responses [18], [19] it probably involves activation of the vagus, which innervates the gastrointestinal tract and exerts a major influence on gastric emptying. It is also possible that unpleasant taste could produce a stress-like response, inhibiting gastric emptying or otherwise reducing gastrointestinal absorption by activating the sympathetic nervous system. An intriguing issue is how taste exposure–lasting only a few seconds–can have effects on blood fat fuels 2 or even 4 h later. One possibility is that taste stimulation activates neural circuitry, probably in the brain (although possibly in the enteric nervous system), that maintains strong but not complete inhibition of gastric emptying or intestinal absorption until the stomach is nearly empty. Alternatively, secondary effects initiated by nervous responses might be involved, such as the modulation of fat trafficking.

There is a growing literature that taste influences fat disposition [20]–[26]. Our results suggest that unpleasant tastes reduce the gastric emptying of fat, leading to lowered concentrations of triglycerides and NEFAs in the blood. The effects of a pleasant sweet taste were less clear, but this does not detract from the main implication of this paper. It may be possible to manipulate the taste of food to mitigate postprandial hypertriglycemia which, in turn, could alter the risk of cardiovascular disease (see introduction).

## References

[1] Miller M, Stone NJ, Ballantyne C, Bittner V, Criqui MH, et al (2011) Triglycerides and cardiovascular disease: a scientific statement from the American Heart Association. Circulation 123: 2292–2333. 21502576 10.1161/CIR.0b013e3182160726

[2] Chapman MJ, Ginsberg HN, Amarenco P, Andreotti F, Borén J, et al (2011) Triglyceride-rich lipoproteins and high-density lipoprotein cholesterol in patients at high risk of cardiovascular disease: evidence and guidance for management. Eur Heart J 32: 1345–1361. 21531743 10.1093/eurheartj/ehr112 PMC3105250

[3] Bansal S, Buring JE, Rifai N, Mora S, Sacks FM (2007) Fasting compared with nonfasting triglycerides and risk of cardiovascular events in women. JAMA 298: 309–316. 17635891 10.1001/jama.298.3.309

[4] Mora S, Rifai N, Buring JE, Ridker PM (2008) Fasting compared with nonfasting lipids and apolipoproteins for predicting incident cardiovascular events. Circulation 118: 993–1001. 18711012 10.1161/CIRCULATIONAHA.108.777334 PMC2574817

[5] Chan DC, Pang J, Romic G, Watts GF (2013) Postprandial hypertriglyceridemia and cardiovascular disease: current and future therapies. Curr Atheroscler Rep 15: 309. 23345190 10.1007/s11883-013-0309-9

[6] Alipour A, Elte JW, van Zaanen HCT, Rietveld AP, Castro Cabezas M (2008) Novel aspects of postprandial lipemia in relation to atherosclerosis. Atheroscler Suppl 9: 39–44. 10.1016/j.atherosclerosissup.2008.05.007 18595782

[7] Flynn FW, Grill HJ, Schwartz GJ, Norgren R (1991) Central gustatory lesions: I. Preference and taste reactivity tests. Behav Neurosci 105: 933–943. 1663764 10.1037//0735-7044.105.6.933

[8] Garcia-Bailo B, Toguri C, Eny KM, El-Sohemy A (2009) Genetic variation in taste and its influence on food selection. OMICS 13: 69–80. 18687042 10.1089/omi.2008.0031

[9] Rosenstein D, Oster H (1988) Differential facial responses to four basic tastes in newborns. Child Dev 59: 1555–1568. 3208567

[10] Steiner JE, Glaser D, Hawilo ME, Berridge KC (2001) Comparative expression of hedonic impact: affective reactions to taste by human infants and other primates. Neurosci Biobehav Rev 25: 53–74. 11166078 10.1016/s0149-7634(00)00051-8

[11] Spector AC, Glendinning JI (2009) Linking peripheral taste processes to behavior. Curr Opin Neurobiol 19: 370–377. 19674892 10.1016/j.conb.2009.07.014 PMC2756742

[12] Valenstein ES, Cox VC, Kakolewski JW (1967) Polydipsia elicited by the synergistic action of a saccharin and glucose solution. Science 157: 552–554. 6028919 10.1126/science.157.3788.552

[13] Inui-Yamamoto C, Yuichi F, Takashi Y (2009) Hedonics of taste influence the gastric emptying in rats. Physiol Behav 96: 717–722. 19385026 10.1016/j.physbeh.2009.01.013

[14] Naim M, Kare MR, Merritt AM (1978) Effects of oral stimulation on the cephalic phase of pancreatic exocrine in dogs. Physiol Behav 20: 563–570. 684091 10.1016/0031-9384(78)90248-2

[15] Powley TL, Berthoud HR (1985) Diet and cephalic phase insulin responses. Am J Clin Nutr 42: 991–1002. 3933326 10.1093/ajcn/42.5.991

[16] Tonosaki K, Hori Y, Shimizu Y, Tonosaki K (2007) Relationships between insulin release and taste. Biomed Res 28: 79–83. 17510492 10.2220/biomedres.28.79

[17] Abdallah L, Chabert M, Louis-Sylvestre J (1997) Cephalic phase responses to sweet taste. Am J Clin Nutr 65: 737–743. 9062523 10.1093/ajcn/65.3.737

[18] Smeets PA, Erkner A, de Graaf C (2010) Cephalic phase responses and appetite. Nutr Rev 68: 643–655. 20961295 10.1111/j.1753-4887.2010.00334.x

[19] Teff KL (2011) How neural mediation of anticipatory and compensatory insulin release helps us tolerate food. Physiol Behav 103: 44–50. 21256146 10.1016/j.physbeh.2011.01.012 PMC3056926

[20] Ramirez I (1985) Oral stimulation alters digestion of intragastric oil meals in rats. Am J Physiol 248: R459–463. 3985188 10.1152/ajpregu.1985.248.4.R459

[21] Mattes RD (2002) Oral fat exposure increases the first phase triacylglycerol concentration due to release of stored lipid in humans. J Nutr 132: 3656–3662. 12468603 10.1093/jn/132.12.3656

[22] Mattes RD (2009) Oral fat exposure pattern and lipid loading effects on the serum triacylglycerol concentration of humans. Chemosens Percept 2: 180–185. 20352072 10.1007/s12078-009-9062-4 PMC2843922

[23] Robertson MD, Mason AO, Frayn KN (2002) Timing of vagal stimulation affects postprandial lipid metabolism in humans. Am J Clin Nutr 76: 71–77. 12081818 10.1093/ajcn/76.1.71

[24] Chavez-Jauregui RN, Mattes RD, Parks EJ (2010) Dynamics of fat absorption and effect of sham feeding on postprandial lipema. Gastroenterol 139: 1538–1548. 10.1053/j.gastro.2010.05.002 PMC2948783 20493191

[25] Mattes RD (2011) Oral fatty acid signaling and intestinal lipid processing: support and supposition. Physiol Behav 105: 27–35. 21324328 10.1016/j.physbeh.2011.02.016

[26] Tittelbach TJ, Mattes RD (2001) Oral stimulation influences postprandial triacylglycerol concentrations in humans: nutrient specificity. J Am Coll Nutr 20: 485–493. 11601563 10.1080/07315724.2001.10719057

[27] Iwatsuki K, Uneyama H (2012) Sense of taste in the gastrointestinal tract. J Pharmacol Sci 118: 123–128. 22293296 10.1254/jphs.11r08cp

[28] Jang HJ, Kokrashvili Z, Theodorakis MJ, Carlson OD, Kim BJ, et al (2007) Gut-expressed gustducin and taste receptors regulate secretion of glucagon-like peptide-1. Proc Natl Acad Sci U S A 104: 15069–15074. 17724330 10.1073/pnas.0706890104 PMC1986614

[29] Margolskee RF, Dyer J, Kokrashvili Z, Salmon KSH, Ilegems E, et al (2007) T1R3 and gustducin in gut sense sugars to regulate expression of Na+-glucose cotransporter 1. Proc Natl Acad Sci U S A 104: 15075–15080. 17724332 10.1073/pnas.0706678104 PMC1986615

[30] Janssen S, Laermans J, Verhulst PJ, Thijs T, Tack J, et al (2011) Bitter taste receptors and α-gustducin regulate the secretion of ghrelin with functional effects on food intake and gastric emptying. Proc Natl Acad Sci U S A 108: 2094–2099. 21245306 10.1073/pnas.1011508108 PMC3033292

[31] Glendinning JI, Yiin YM, Ackroff K, Sclafani A (2008) Intragastric infusion of denatonium conditions flavor aversions and delays gastric emptying in rodents. Physiol Behav 93: 757–765. 18174110 10.1016/j.physbeh.2007.11.029 PMC2396241

[32] Wicks D, Wright J, Rayment P, Spiller R (2005) Impact of bitter taste on gastric motility. Eur J Gastroenterol Hepatol 17: 961–965. 16093874 10.1097/00042737-200509000-00012

[33] Robertson MD, Parkes M, Warren BF, Ferguson DJ, Jackson KG, et al (2003) Mobilisation of enterocyte fat stores by oral glucose in humans. Gut 52: 834–839. 12740339 10.1136/gut.52.6.834 PMC1773679

[34] Bartoshuk LM (1977) Water taste in mammals. In: Weijnen JAWM, Mendelson J, editors. Drinking behavior: oral stimulation, reinforcement and preference. New York: Plenum Press. 317–339.

[35] Robertson MD, Henderson RA, Vist GE, Rumsey RDE (2002) Extended effects of evening meal carbohydrate-to-fat ratio on fasting and postprandial substrate metabolism. Am J Clin Nutr 75: 505–510. 11864856 10.1093/ajcn/75.3.505

